# Stability mechanism and countermeasure of the solid coal rib in deep gob-side entry retaining: Insights from theoretical analysis numerical simulation

**DOI:** 10.1016/j.heliyon.2024.e24174

**Published:** 2024-01-11

**Authors:** Shiqiang Xu, Zizheng Zhang, Jinlin Xin, Jianbiao Bai, Xingen Ma, Ren Zhai, Wenda Wu

**Affiliations:** a Work Safety Key Lab on Prevention and Control of Gas and Roof Disasters for Southern Goal Mines, Hunan Provincial Key Laboratory of Safe Mining Techniques of Coal Mines, Hunan University of Science and Technology, Xiangtan 411201, China; b State Key Laboratory of Coal Resources and Safe Mining, China University of Mining and Technology, Xuzhou 221116, China; c School of Mining and Geology, Xinjiang Institute of Engineering, Urumqi 830023, China; d Huaneng Coal Technology Research Co., China Huaneng, Beijing 100070, China; e Yankuang Energy Group CO., LTD. Jing NO.3 Coal Mine, Jining 272004, China; f College of Mining Engineering, Taiyuan University of Technology, Taiyuan 030024, China

**Keywords:** Gob-side entry retaining, Solid coal rib failure, Plastic state, Control countermeasure

## Abstract

The stability and integrity of the solid coal rib in deep gob-side entry retaining (GER) can be compromised due to the cyclic loading and unloading caused by mining-induced stress. This can lead to failure of the deep GER during depressurized mining operations. In this study, we focus on a specific case at the 94103 working face in Qishan Coal Mine of Xuzhou Mining Bureau. We establish an engineering model that describes the interaction between the solid coal rib and the main roof in GER, aiming to elucidate the characteristics of main roof failure and instability throughout the entire GER process. this model particularly emphasizes the mechanical properties of the solid coal rib as a contributing factor. Additionally, developed a limit stress state model for evaluating bolt-supported plastic solid coal ribs, which helps determine appropriate support resistance levels to prevent two common forms of failure in these ribs. Furthermore, created a numerical calculation model to investigate different bolt conditions' impact on solid coal rib failure mechanisms. Finally, based on field monitoring data validation, we propose control measures for reinforcing solid coal ribs along with suggestions for roof support design and filling body construction schemes under similar geological conditions. These research findings offer valuable guidance for developing effective reinforcement strategies for filling bodies.

## 1 Introduction

The implementation of gob-side entry retaining (GER) technology provides technical support for the successful execution of deep continuous pressure relief mining and deep coal and gas mining. It achieves this by reducing the necessity for extensive roadway excavation, mitigating mining tension, enhancing resource recovery efficiency, eliminating stress concentration on coal pillars, and addressing the issue of gas accumulation in upper corners [[1], [2], [3], [4], [5], [6]]. Over time, GER technology has been progressively applied across various coal seam thicknesses - from thin seams (less than 1.3 m) [[7], [8], [9]], medium-thick seams (1.3 m–3.5 m) [[10], [11], [12]], fully mechanized thick seams (more than 3.5 m) [[13], [14], [15]], to even fully mechanized top coal caving mining operations [16,17]. Notably, this technology has been successfully employed at depths exceeding 1000 m [18,19].

According to Xie et al. [[20], [21], [22], [23], [24], [25]], it has been observed that with increasing mining depth, the coal seam initially reaches its failure strength and plastic limit due to its low strength and stiffness. Subsequently, a brittle plastic transformation occurs, resulting in spalling, impact, and significant plastic deformation of the coal seam during mining operations. The surrounding rock in deep roadways exhibits distinct characteristics such as deterioration of mechanical properties, transition from brittle to plastic deformation, and nonlinear large deformation. In the case of a deep coal miner's goaf edge roadway (GER), the formation, development, movement, and stress transfer of lateral fracture structures contribute to gradual solid coal rib failure throughout the GER process. This leads to extensive plastic deformation of the rib. The substantial deformations and failures observed in retained roadways' solid coal ribs are attributed to expansion of their plastic zones induced by mining stresses transmitted through lateral fracture structures [[26], [27], [28]].

In recent years, numerous scholars worldwide have made significant advancements in comprehending the structural characteristics of coal mine roof failures [[29], [30], [31], [32]]. Chen [33] emphasized that the initial rupture of the primary roof occurs at the solid coal rib side and subsequently twice at the filling body side. Ma et al. [34] summarized a four-stage evolutionary process for the stress state of surrounding rocks: supporting stress, roof cutting stress, pre-mining stress, and post-mining stress. These findings substantiate the single plane theory and offer valuable insights for designing cutting angles under pre-mining stress conditions. Tian et al. [9] firstly identified challenges and key factors in controlling surrounding rock behavior when retaining gob-side entries with soft roofs, floors, and seams in thin coal seams. They developed a mechanical model to analyze interactions between roadside backfill bodies and roofs in such scenarios. To investigate failure mechanisms near gob edges caused by main roof deformation, Li et al. [35] constructed a UDEC Trigon model incorporating robust and thick hanging roofs.

In order to ensure the stability of the solid coal rib in GER, extensive research has been conducted by scholars and engineers. Yu [36] utilized dynamic simulation methods to optimize the grouting time of the solid coal rib. They proposed a combined approach of anchor injection reinforcement to repair crushed surrounding rock along the mining process for coal refactoring. Meng et al. [37] performed numerical simulations using UDEC software's Trigon model to analyze fracture propagation and distribution in the roadside backfill body under both static and dynamic loadings. The results indicated that static loading played a more significant role in causing failure compared to dynamic loading. Ma et al. [38] suggested strengthening roof support measures to maintain top coal stability, as well as reinforcing the support structure of coal ribs for ensuring gob-side entry stability with top coal intact. Su et al. [16] on their part, put forward roadway support technology involving strong rib and controlled roof in GER, emphasizing that enhancing the strength of solid coal ribs could prevent premature roof breakage above them. These studies demonstrate an understanding of utilizing high-strength bolting techniques and solid entity coal ribs for effective control over surrounding rock integrity, which holds significant implications for practical applications.However, there remains a gap in research regarding load-bearing characteristics and failure mechanisms specific to solid coal ribs in GER, as well as limited knowledge about their interaction with bolt-net support systems when subjected to plastic deformation or their stability during such conditions.

This paper presents a case study of GER engineering practice in Qishan Coal Mine, focusing on the analysis of the failure mechanism of the coal rib during the main roof breaking process. An interaction mechanical model between the solid coal rib and the main roof in GER is established to investigate this mechanism. Furthermore, numerical calculation models are developed to assess the stability of the lateral block structure under different bolt conditions considering the mechanical properties of the solid coal rib. Finally, a stability control principle for managing plastic state behavior of solid coal ribs in GER is proposed as a basis for ensuring surrounding rock system stability in deep Coal Mines.

## 2 Geological and mining conditions

The case study focused on the geological and mining conditions of the 94103 working face in the Qishan Coal Mine, which falls under the jurisdiction of Xuzhou Mining Bureau in China. The 94103 working face was extracted from No.9 coal seam, with a haulage gate retaining the roadway along the gob. The dip angle of No.9 coal seam is 7°, and it has a thickness of 2.1 m. The average depth at which the working face is buried is approximately 800 m, while its length measures around 150 m. In terms of strata composition, there exists sandy mudstone as an immediate roof above the working face, with a thickness of about 6.7 m. This particular type of mudstone possesses characteristics such as high density, soft texture, and flat fracture surface. As for the main roof, it primarily consists of sandstone interbedded with mudstone layers that have a total thickness of approximately 14.3 m; beneath this upper sandstone layer lies additional sporadic bands of sandstone within mudstone. Regarding floor conditions within this area, both immediate and main floors consist primarily of mudstone layers measuring approximately 1.8 m and 8.2 m thick respectively. To facilitate transportation operations within this mine section, a haulage gate was excavated along the roof level adjacent to No 0.9 coal seam; initially measuring dimensions at width by height (4.5 m x 2.l m) before undergoing GER (Ground Engagement Replacement) treatment resulting in its current reduced width to about 3.6 m wide.

Regarding original roadway support parameters implemented in Qishan Coal Mine's haulage gate labeled as 94103, ultra-high strength bolts having a diameter of 16 mm and length extending up to 2000 mm were chosen to serve as both roof and rib support; these bolts were spaced apart at intervals measuring 800 mm × 800 mm across rows. Additionally, a single row consisting anchor cables having a diameter of 15.24 mm and length of 7 m were arranged in the middle with spacing measuring approximately 3.2 m.

## 3 Stability analysis of main roof during the Whole GER process

### 3.1 Interaction mechanical model between the solid coal rib and the main roof in GER

**Fig. 1:**
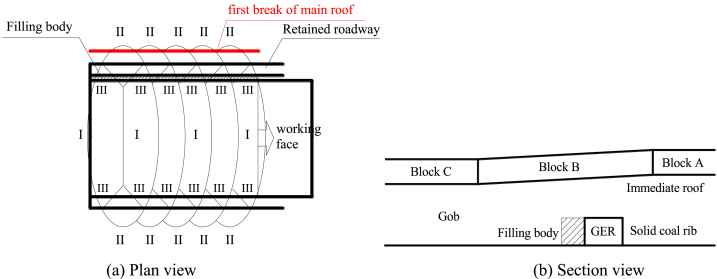
First break of the main roof.

**Fig. 2:**
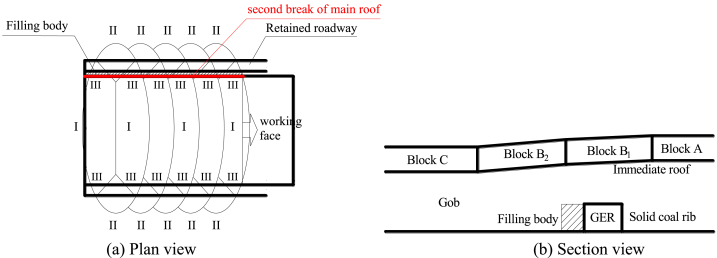
Second break of the main roof.

Based on numerous field practices in GER [10,16,17], it has been observed that: (i) The impact of the overlying strata beneath the primary key stratum on the main roof (sub-key stratum) is significantly more significant than that of roadway support; (ii) The construction of the filling body along gob side can only occur at a specific distance behind the working face and often faces challenges in achieving complete connection with the immediate roof. Its supportive role becomes effective only when there is a certain degree of roof subsidence, indicating its passive nature. (iii) In comparison to solid coal rib's supporting effect, the filling body along gob side exhibits smaller strength and width, with negligible influence on initiating first breakage of the main roof (refer to Fig. 1); (iv) Once roof subsidence reaches a particular threshold and high support strength is achieved by filling body along gob side, double breakage may occur above this structure along its side (refer to Fig. 2). Therefore, we can divide GER's failure process into two stages: initial breakage and subsequent secondary breakage.

**Fig. 3:**
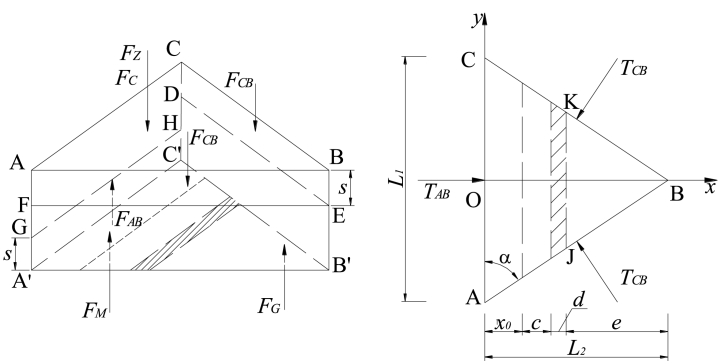
Diagram illustrating the stress distribution in key block B prior to the second fracture.

According to Fig. 1, the force diagram of key block B prior to the second break is illustrated in Fig. 3. Additionally, several assumptions have been made for the interaction mechanical model [33].

(i) Key block B is in contact with both front and rear key block C as well as lateral key block A. The relationship between these blocks is considered a plastic hinged connection.
(ii) The forces exerted by key block C on key block B before and after are assumed to be equal. These forces, denoted as *F*_*CB*_ and *T*_*CB*_ respectively, act uniformly at positions FE and DE while also acting at s/2 vertically.
(iii) Similarly, the resultant of vertical shear force and horizontal thrust force exerted by lateral key block A on key block B is represented by *F*_*AB*_ and *T*_*AB*_ respectively. These forces act uniformly at position GH while also acting at s/2 in the height direction.
(iv) It should be noted that when considering movement, both key block B and the soft rock layer above it move together as a unit load on top of key block B. This combined weight is represented by F_Z_ which acts upon the center of gravity of key block B.
(v) Prior to the second breakage event of key lock B, there is no gangue supporting force present within goaf area *F*_*G*_ on top of this particular section.
(vi) Furthermore, it can be assumed that immediate roof above roadway moves synchronously with main roof towards gob side; thus, ignoring any impact caused by breaking angle from immediate roof strata. The weight associated with immediate roof can be represented as *F*_*H*_ which acts upon center point ACJK within trapezoidal shape.
(vii) Key Block B rotates towards gob side along rotation axis GH with an angle *θ.*
(viii) Lastly FC represents combined mining abutment pressure from working face; however, when not influenced by advance abutment pressure from working face then *F*_*C*_ equals zero.

#### 3.1.1 Supporting force of the solid coal rib (*F*_*S*_)

In the vicinity of the initial operational area, the superficial section of the solid coal rib typically exhibits plastic behavior, and its load-bearing capacity is determined using the subsequent equation.

(1) $$\sigma_{m}=\left(\frac{C_{0}}{\tan\;\varphi_{0}}+\frac{p_{x}}{A}\right)e^{\frac{2\;\tan\;\varphi_{0}}{h_{d}\cdot A}\cdot \left(x_{0}-x\right)}-\frac{C_{0}}{\tan\;\varphi_{0}}$$

In Eq. (1), *h*_*d*_ represents the roadway height; *A* denotes the lateral pressure coefficient; *K* signifies the stress concentration coefficient; *γ* represents the average bulk density of overburden strata; *H* indicates the buried depth of coal seam; *C*_0_ refers to the cohesive force of solid coal; *φ*_0_ represents the friction of solid coal; and *p*_*x*_ denotes the support strength of solid coal rib.

Therefore, it can be inferred that the supporting force exerted by the solid coal rib on the immediate roof can be expressed as follows:

(2) $$F_{S}=\int_{0}^{x_{0}}\sigma_{m}\left[\frac{-2}{\tan\;\alpha }\left(x-L_{2}\right)\right]dx$$

In Eq. (2), *α* represents the angle at the base of key block B and can be calculated using the formula *α* = arctan (2*L*_2_/*L*_1_). Here, *L*_2_ denotes the length of key block B, while *L*_1_ refers to the distance between the main roof and its weight. Moreover, *x*_0_ designates the fracture position of key block B situated above the solid coal rib.

(3) $$\begin{array}{l} L_{2}=\frac{2L_{1}}{17}\left[\sqrt{{\left(10\frac{L_{1}}{L_{m}}\right)}^{2}+102}-10\frac{L_{1}}{L_{m}}\right]\\ x_{0}=\frac{h_{d}A}{2\;\tan\;\varphi_{0}}\ln\left(\frac{K\gamma H+\frac{C_{0}}{\tan\;\varphi_{0}}}{\frac{C_{0}}{\tan\;\varphi_{0}}+\frac{p_{x}}{A}}\right) \end{array}\}$$

In Eq. (3), *Lm* is the working face length.

#### 3.1.2 2 supporting force of the filling body (FR)

The supporting force for the filling body can be calculated using the formula:

(4) $$F_{R}=\int_{x_{0}+c}^{x_{0}+c+d}\sigma_{d}\left[\frac{-2}{\tan\;\alpha }\left(x-L_{2}\right)\right]dx$$

In Eq. (4), *c* represents the width of the retained roadway; *d* denotes the width of the filling body; *σd* indicates the uniaxial compressive strength of the solidified filling material.

#### 3.1.3 Gangue supporting force in the gob on key block B (F_G_)

The supporting force of the gangue in key block B can be determined using the following equation:

(5) $$F_{G}=\int_{a_{0}}^{L_{2}\;\cos\;\theta }f_{G}\left(-\frac{2}{\tan\;\alpha }\left(x-L_{2}\right)\right)dx$$

In Eq. (5), *θ* represents the rotation angle of key block B, *a*0 denotes the abscissa of the point where key block B just touches the gangue, and *fG* indicates the supporting force generated by gangue per unit area.

(6) $$\begin{array}{l} f_{G}=K_{G}\left(S_{0}+x\;\sin\;\theta -\left\{m-\left[m\left(1-\eta \right)K_{m}+H_{l}\left(K_{l}-1\right)\right]\right\}\right)\\ \theta_{0}=\arcsin\left(\frac{m-\left[m\left(1-\eta \right)K_{m}+H_{l}\left(K_{l}-1\right)\right]-S_{0}}{x}\right) \end{array}\}$$

In Eq. (6), *η* stands for the recovery rate of the working face; *m* represents coal seam height; *Km* and *Kl* are respectively referred to as crushing expansion coefficients for coal and immediate roof; *Hl* signifies immediate roof height; *KG* is defined as support coefficient for caving gangue, measured in MPa/m; *S*0 refers to subsidence of main roof at fracture.

#### 3.1.4 Supporting force of the immediate roof (F_M_)

**Fig. 4:**
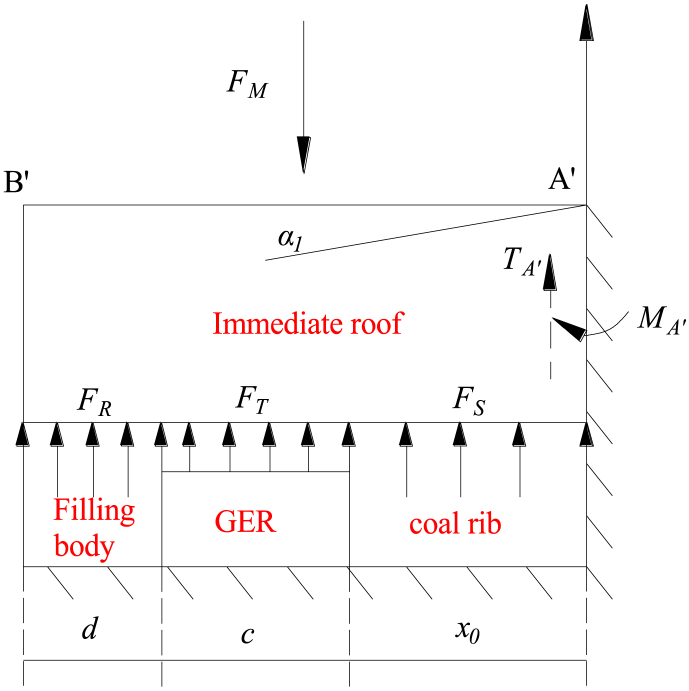
Mechanical model of the immediate roof in GER.

Due to its significantly higher stiffness compared to the immediate roof and coal seam, key block B exerts a 'given deformation' effect on the underlying coal and rock. Disregarding the impact of the breaking angle of the immediate roof, Fig. 4 presents a simplified mechanical model illustrating GER's immediate roof. The upper boundary bears the reaction load from key block B, while the lower boundary experiences both roadway support force and coal rib support force. The right boundary represents a free surface, whereas the left boundary is simplified as a simply supported boundary with bending moment and shear force.

The moments exerted by *F*_*H*_, *F*_*S*_, *F*_*M*_, *F*_*R*_, *F*_*T*_ and *F*_*G*_ on the rotation axis GH of key block B can be calculated as follows:

(7) $$\begin{array}{l} R_{H}={\left(x_{0}+c+d\right)}^{2}L_{1}\gamma_{l}H_{l}/4\\ R_{S}=\int_{0}^{x_{0}}\sigma_{m}\left(-\frac{2}{\tan\;\alpha }\left(x-L_{2}\right)\right)xdx\\ R_{M}=F_{M}\left(x_{0}+c+d\right)/2\\ R_{R}=\int_{x_{0}+c}^{x_{0}+c+d}\sigma_{d}\left(-\frac{2}{\tan\;\alpha }\left(x-L_{2}\right)\right)xdx\\ R_{T}=\int_{x_{0}}^{x_{0}+c}f_{T}\left(-\frac{2}{\tan\;\alpha }\left(x-L_{2}\right)\right)xdx\\ R_{G}=\int_{a_{0}}^{L_{2}\;\cos\;\theta }f_{G}\left(-\frac{2}{\tan\;\alpha }\left(x-L_{2}\right)\right)xdx \end{array}\}$$

In Eq. (7), *γ*_*l*_ represents the density of the immediate roof; *f*_*T*_ denotes the strength of support in the retained roadway.

In case a fixed boundary condition is applied to end A′ for the immediate roof,

(8) $$M_{A^{\prime }}=\frac{1}{6}\sigma_{tl}{H_{l}}^{2}L_{1}$$

In Eq. (8), *σtl* represents its tension.

The net moment generated by each force around rotating axis GH is determined to be zero. Consequently, it can be derived in the following manner:

(9) $$F_{M}=\frac{2\left(R_{S}+R_{T}+R_{R}+M_{A^{\prime }}-R_{H}\right)}{x_{0}+c+d}$$

#### 3.1.5 Horizontal thrust of the key block B (TAB, TCB)

**Fig. 5:**
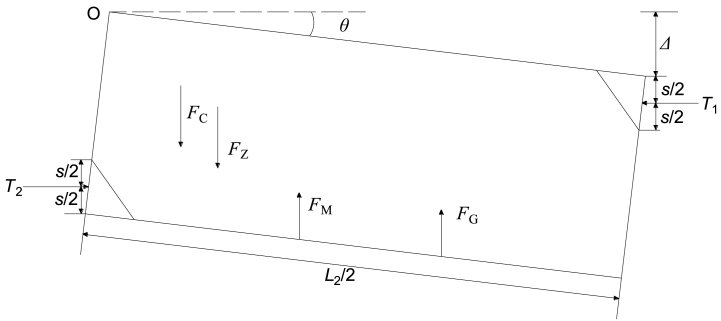
State of the key block B when rotating and sinking.

The condition of key block B during rotation and subsidence is illustrated in Fig. 5. By considering the equilibrium of moments at point O, the following equation can be derived:

(10) $$-T_{1}\left(\Delta +\frac{s}{2}\right)+T_{2}\left(H_{E}-\frac{s}{2}\right)+R_{M}+R_{G}-F_{Z}\frac{L_{2}}{3}=0$$

In Eq. (10), *F*_Z_ represents the weight of key block B and its overlying soft rock layer.

(11) $$F_{Z}=\left(h_{Z}+H_{E}\right)\gamma_{Z}L_{1}L_{2}/2$$

In Eq. (11), *h*_*Z*_ denotes the height of this overlying layer, and *γ*_*Z*_ signifies its bulk density.

Consequently, the horizontal thrust exerted by key block A on key block B (*T*_*AB*_) can be calculated using the following Eq. (12):

(12) $$T_{AB}=\frac{4F_{Z}L_{2}-12\left(R_{M}+R_{G}\right)}{3\left(2H_{E}-L_{2}\;\sin\;\theta \right)}$$

Similarly, for determining the horizontal thrust exerted by key block C on key block B (*T*_*CB*_), a similar approach is employed:

(13) $$T_{CB}=\frac{2F_{Z}L_{2}-6\left(R_{M}+R_{G}\right)}{3\left(2H_{E}-L_{2}\;\sin\;\theta \right)\cos\;\alpha }$$

#### 3.1.6 Shear force of the key block B (FAB, FCB)

The resultant moment of each force to the rotating axis GH is zero. Hence, it can be obtained as follows:

(14) $$2T_{CB}\left(H_{E}-s-\frac{L_{2}\;\sin\;\theta }{2}\right)\cos\;\alpha +R_{M}+R_{G}-\;2F_{CB}\;\cos\;\theta \frac{L_{2}}{2}-F_{Z}\;\cos\;\theta \frac{L_{2}}{3}=0$$

That is,

(15) $$F_{CB}=\frac{T_{CB}\;\cos\;\alpha }{L_{2}\;\cos\;\theta }\left(H_{E}-\frac{L_{2}\;\sin\;\theta }{2}\right)+\frac{R_{M}+R_{G}}{L_{2}\;\cos\;\theta }-\frac{F_{Z}}{3}$$

The resultant force in the vertical direction from key plate B is 0, it can be obtained as follows:

(16) $$F_{AB}=2F_{CB}+F_{Z}-F_{M}-F_{G}$$

#### 3.1.7 Bending moment of the key block B due to the second break above the filling body (M0)

**Fig. 6:**
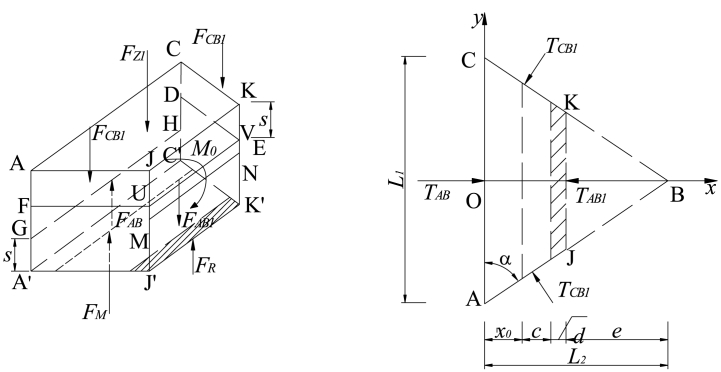
Stress diagram of the key block B_1_.

Assuming that the key block B reaches the maximum tensile stress above the outside of the filling body, it is cut off to form key blocks B_1_ and B_2_. Fig. 6 shows the separate the key block B_1_ for mechanical analysis.

As shown in Fig. 6, setting the scale coefficient *k*_0_ = *e*/*L*_2_, the forces of trapezoidal ACJK are as follows:

(17) $$\begin{array}{l} T_{AB1}=\left(1-k_{0}\right)T_{AB}，T_{CB1}=\left(1-k_{0}\right)T_{CB}\\ F_{CB1}=\left(1-k_{0}\right)F_{CB}，F_{Z1}=\left(1-k_{0}^{2}\right)F_{Z} \end{array}\}$$

The resultant moment of each force to the rotating axis GH is zero. Hence, it can be obtained as follows:

(18) $$\begin{array}{l} R_{M}-F_{Z1}\left(x_{0}+c+d\right)/2+2\left(1-k_{0}\right)T_{CB}\;\cos\;\alpha \left[H_{E}-s-\frac{1}{2}\left(L_{2}-e\right)\sin\;\theta \right]-\\ 2F_{CB1}\;\cos\;\theta \frac{L_{2}-e}{2}--F_{AB1}\left(L_{2}-e\right)\cos\;\alpha -T_{AB1}\left(\frac{H_{E}}{2}-s\right)\cos\;\alpha -M_{0}=0 \end{array}$$

That is,

(19) $$\begin{array}{l} M_{0}=\left(1-k_{0}^{2}\right)F_{Z}\left(x_{0}+c+d\right)/2+2\left(1-k_{0}\right)T_{CB}\;\cos\;\alpha \left[H_{E}-s-\frac{1}{2}\left(L_{2}-e\right)\sin\;\theta \right]-\\ 2\left(1-k_{0}\right)F_{CB}\;\cos\;\theta \frac{L_{2}-e}{2}-F_{AB1}\left(L_{2}-e\right)\cos\;\alpha -T_{AB1}\left(\frac{H_{E}}{2}-s\right)\cos\;\alpha +R_{M} \end{array}$$

Where, *TAB1* and *FAB1* are the horizontal thrust and shear force on key block B_1_ before cutting off key block B_2_, and the action point is located at half of the thickness of key block B.

#### 3.1.8 Horizontal thrust and shear force on key block B_1_ (TAB1, FAB1)

**Fig. 7:**
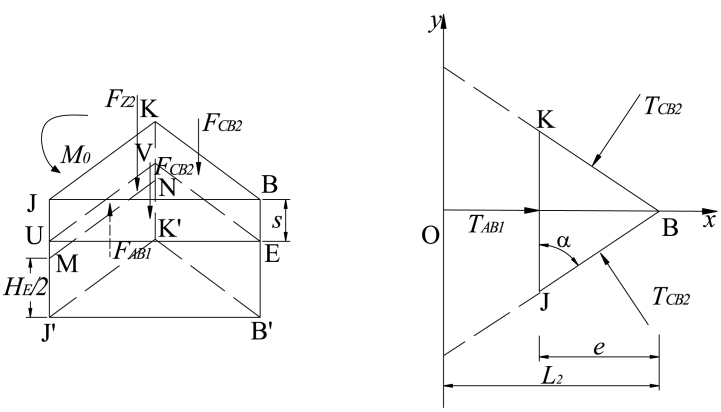
Stress diagram of the key block B_2_.

The balance method is used to solve *TAB1* and *FAB1*, as shown in Fig. 7.

(20) $$\begin{array}{l} \sum F_{z}=F_{AB1}+F_{G}-2F_{CB2}-F_{Z2}=0\\ \sum F_{x}=T_{AB1}-2T_{CB2}\;\cos\;\alpha =0 \end{array}\}$$

That is,

(21) $$\begin{array}{l} F_{AB1}=k_{0}\left(2F_{CB}-k_{0}F_{Z}\right)-F_{G}\\ T_{AB1}=2k_{0}T_{CB}\;\cos\;\alpha \end{array}\}$$

### 3.2 Influence law of second lateral break position and structural morphology of the main roof

Considering the widely accepted belief that the initial rupture of the primary roof occurs in front of the operational area, this analysis primarily focuses on subsequent breakage. Based on engineering practices observed at working face 94103 in Qishan Coal Mine, Xuzhou Mining Bureau, several parameters were noted: a retained roadway width of 3.6 m, filling body width of 1.2 m, *H* = 800 m, *m* = 2.1 m, *h*_*d*_ = 2.8 m, *A* = 0.4, *C*_0_ = 2.0 MPa, *φ*_0_ = 35°, *γ* = 24 kN/m^3^, *K* = 2, *H*_*E*_ = 14 m, *H*_*R*_*=*86 m, *σ*_*d*_ = 13.5 MPa, *h*_*Z*_ = 6 m, *H*_*l*_ = 6 m, *σ*_*c*_ = 35 MPa, *θ* = 3°, *f*_T_ = 0.2 MPa, *R*_*tl*_ = 0.5 MPa, *K*_*G*_ = 2.0 MPa/m, *K*_*m*_ = 1.05, *K*_*l*_ = 1.1, *η* = 0.95, *S*_0_ = 0.

**Fig. 8:**
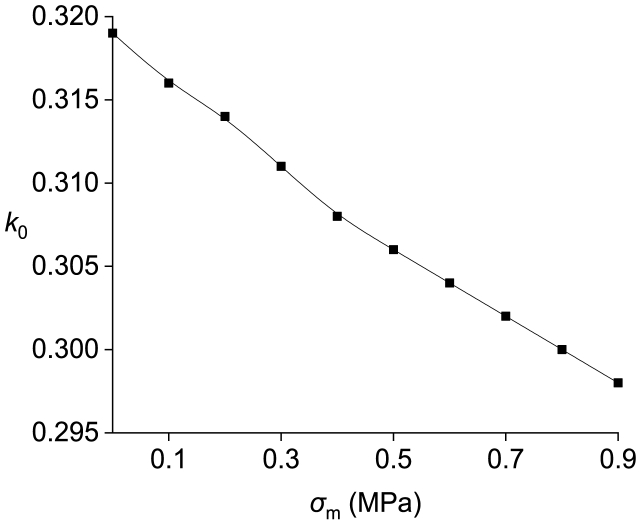
Relationship between the support resistance of the solid coal rib (*σ*_*m*_) and the second break position of the main roof (*k*_*0*_).

**Fig. 9:**
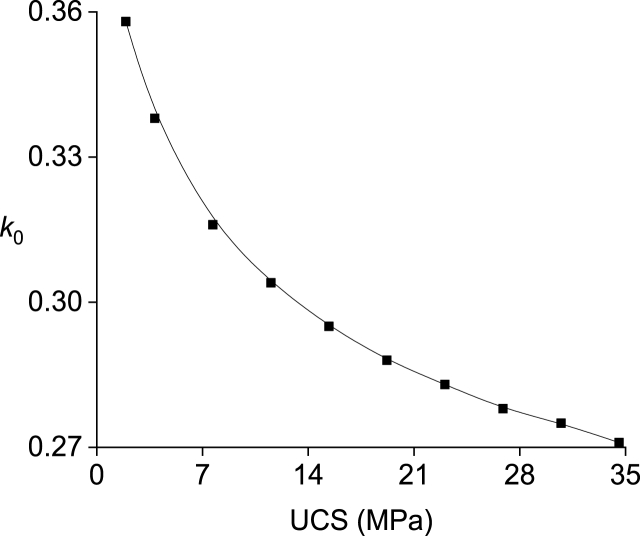
Relationship between the UCS of the solid coal rib and the second break position of the main roof (*k*_*0*_).

The correlation between the support resistance of the solid coal rib (with a reference value of 0.1 MPa) and the second break position of the main roof (k0) is illustrated in Fig. 8. The relationship between the second break position of the main roof (k0) and the uniaxial compressive strength of the solid coal rib (with a reference value of 7.684 MPa) is depicted in Fig. 9.

It is apparent that as the mechanical properties of the solid coal rib improve, there is a gradual shift in the second break position of the key block of the main roof away from the solid coal rib. In other words, this break position tends to be closer to the filling body and gob. 10.13039/100014337Furthermore, an increase in support resistance provided by the solid coal rib results in a gradual displacement of the second break position of the key block from its proximity to said rib.

### 3.3 Stability analysis of the key block of the main roof in GER

According to the theory of masonry beam, the failure and instability of key block B are influenced by key blocks A and C. It is imperative to prevent sliding instability of key block B at small angles and extrusion deformation instability as the angle increases.

#### 3.3.1 Sliding instability of key block B

In order to prevent the key block B from sliding and instability along the roadway side, the conditions as follows must be meet.

(22) $$T_{AB}\;\tan\left(\varphi -\beta \right)>F_{AB}$$

In Eq. (22), the sliding instability coefficient (*K*_1_) is defined to express the possibility of the sliding and instability.

(23) $$K_{1}=\frac{F_{AB}}{T_{AB}\;\tan\left(\varphi -\beta \right)}$$

In Eq. (23), *φ* is the friction angle between the contact surfaces of rock blocks; *β* is the break angle of rock stratum.

#### 3.3.2 Extrusion deformation instability of key block B

In order to prevent the key block B from extrusion deformation instability, the conditions as follows must be meet.

(24) $$T_{AB}<L_{1}s\psi \sigma_{c}$$

In Eq. (24), the sliding instability coefficient (*K*_1_) is defined to express the possibility of the sliding and instability.

(25) $$K_{2}=\frac{T_{AB}}{L_{1}s\psi \sigma_{c}}$$

In Eq. (25), *σ*_*c*_ is the compressive strength of key blocks; *ψ* is the contact coefficient between key blocks.

Consequently, if both *K*_1_ and *K*_2_ are less than 1, the likelihood of key block B experiencing instability is low. The stability of key block B improves as the values of *K*_1_ and *K*_2_ decrease. When *K*_1_ = 1 or *K*_2_ = 1, key block B is in critical instability state. If *K*_1_ < 0 and *K*_2_ < 0, *T*_*AB*_ < 0 indicates that key block B does not need key block a to provide horizontal force, *T*_*AB*_ can maintain balance, and key block B is in a stable state.

According to the GER engineering practice of the 94103 working face in Qishan Coal Mine of Xuzhou Mining Bureau, *c* = 3.6 m, *d* = 1.2 m, *H* = 800 m, *m* = 2.1 m, *h*_*d*_ = 2.8 m, *A* = 0.4, *C*_0_ = 2.0 MPa, *φ*_0_ = 35°, *γ* = 24 kN/m^3^, *K* = 2, *H*_*E*_ = 14 m, *H*_*R*_*=*86 m, *h*_*Z*_ = 6 m, *H*_*l*_ = 6 m, *σ*_*c*_ = 35 MPa, *θ* = 3°, *f*_T_ = 0.2 MPa, *R*_*tl*_ = 0.5 MPa, *K*_*G*_ = 2.0 MPa/m, *K*_*m*_ = 1.05, *K*_*l*_ = 1.1, *η* = 0.95, *S*_0_ = 0, and the single variable method is used to calculate the instability coefficient of key blocks of the main roof in GER (*K*_1_, *K*_2_). Therefore, the relevant influence factors include roadway side support force *σ*_*d*_ (reference value is 13.5 MPa), width of the filling body *d* (reference value is 1.2 m), mechanical properties of solid coal rib *p*_*x*_ (support resistance, reference value is 0.1 MPa, UCS reference value is 7.684 MPa), roadway height *h*_*d*_ (reference value is 2.8 m), buried depth *H* (reference value is 800 m), roadway width *c* (reference value is 3.6 m), immediate roof thickness *H*_*l*_ (reference value is 6.0 m), coal seam thickness *m* (reference value is 2.1 m). Table 1 shows the relationship between the instability coefficient of key blocks of the main roof in GER and its influence factors.

**Table 1:** Relationship between the instability coefficient of key blocks of the main roof in GER and its influence factors.

*σ*_*d*_ (MPa)	1	4	7	10	13.5	16	19	21	24
*K*_1_	1.368	1.157	0.896	0.567	0.052	−0.449	−1.291	−2.093	−3.989
*K*_2_	0.373	0.338	0.302	0.267	0.226	0.196	0.161	0.137	0.102
*p*_*x*_ (MPa)	0.0	0.1	0.2	0.3	0.4	0.5	0.6	0.7	0.8
*K*_1_	0.065	0.052	0.04	0.031	0.023	0.016	0.011	0.006	0.001
*K*_2_	0.225	0.226	0.227	0.228	0.229	0.23	0.231	0.232	0.233
*D* (m)	0.5	1.0	1.5	2.0	2.5	3.0	3.5	4.0	
*K*_1_	0.913	0.347	−0.53	−2.381	−11.211	14.546	5.949	4.157	
*K*_2_	0.32	0.253	0.184	0.112	0.037	−0.039	−0.116	−0.195	
*h*_*d*_ (m)	1.9	2.2	2.5	2.8	3.1	3.4	3.7	4.0	
*K*_1_	0.147	0.121	0.089	0.052	0.007	−0.045	−0.105	−0.174	
*K*_2_	0.251	0.243	0.235	0.226	0.216	0.207	0.197	0.186	
*H* (m)	300	400	500	600	700	800	900	1000	1100
*K*_1_	0.235	0.214	0.183	0.145	0.101	0.052	−0.003	−0.062	−0.127
*K*_2_	0.259	0.252	0.245	0.238	0.232	0.226	0.22	0.214	0.207
*c* (m)	2.0	2.4	2.8	3.2	3.6	4.0	4.4	4.8	5.2
*K*_1_	−0.229	−0.163	−0.095	−0.023	0.052	0.13	0.211	0.295	0.381
*K*_2_	0.255	0.247	0.24	0.232	0.226	0.22	0.214	0.209	0.205
*H*_*l*_ (m)	1	2	3	4	5	6	7	8	9
*K*_1_	0.217	0.19	0.157	0.122	0.086	0.052	0.017	−0.018	−0.058
*K*_2_	0.377	0.321	0.281	0.253	0.236	0.226	0.22	0.217	0.215
*m (m)*	1.2	1.5	1.8	2.1	2.4	2.7	3.0	3.3	3.6
*K*_1_	−0.121	0.022	0.042	0.052	0.116	0.21	0.268	0.286	0.284
*K*_2_	0.178	0.214	0.221	0.226	0.273	0.424	0.761	1.384	2.41
UCS (MPa)	1.921	3.842	7.684	11.53	15.37	19.21	23.05	26.89	30.74
*K*_1_	0.081	0.054	0.052	0.063	0.076	0.087	0.098	0.107	0.115
*K*_2_	0.202	0.213	0.226	0.235	0.241	0.246	0.25	0.253	0.256

As seen from Table 1, the key block B is mainly due to extrusion deformation instability.

(i) The stability of key block B is enhanced by a thicker immediate roof, resulting in reduced values for both *K*_1_ and *K*_2_.
(ii) An increase in the width of the filling body improves the stability of key block B, as evidenced by a decrease in both *K*_1_ and *K*_2_.
(iii) A higher support strength of the filling body contributes to improved stability of key block B, leading to decreased values for both *K*_1_ and *K*_2_.
(iv) The stability of key block B is promoted by an enhanced support strength of solid coal rib, preventing sliding deformation and resulting in a decrease in *K*_1_.
(v) In thick coal seam working faces, maintaining roadway along the gob side becomes challenging due to a significant increase in *K*_2_.
(vi) Retaining roadway along the gob side with large section width proves difficult, indicated by a sharp increase in *K*_1_. There exists an upper limit for roadway section width along the gob side.
(vii) Both *K*_1_ and *K*_2_ are influenced by the uniaxial compressive strength of solid coal rib. Initially, increasing this strength leads to a decrease in *K*_1_ followed by an eventual increase. Conversely, there is a positive correlation between uniaxial compressive strength and value for *K*_2_. This suggests that there exists a certain threshold for supporting strength provided by solid coal rib.

Therefore, ensuring the stability of key block B at the end of the working face during roadway retention along the gob is crucial to prevent excessive stress and potential crushing of the filling body. To enhance the stability of key block B in GER, several measures can be considered.

(i) Utilizing a high active support force to reinforce and increase resistance in the roadway.
(ii) Prioritizing the use of high-strength, high stiffness, and preloaded bolts and anchor cables to strengthen the coal rib before any main roof breakage occurs along it. This will help reduce plastic zone width and create a three-dimensional stress state for increased support force on the coal rib.
(iii) Opting for new materials with rapid resistance increasing speed and high early strength for side filling in roadways. Additionally, promptly sealing and connecting the roof will effectively provide support while maintaining its integrity.

## 4 Stability mechanism of plastic solid coal rib in deep GER

### 4.1 Stability analysis of plastic solid coal rib supported by bolt (cable) in deep GER

**Fig. 10:**
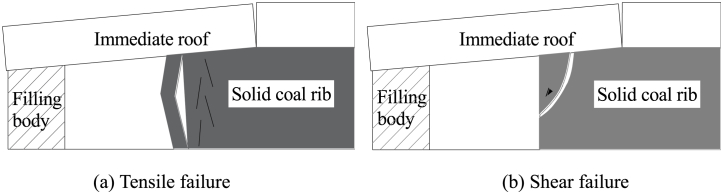
Failure forms of the solid coal rib in GER.

Under the influence of roof pressure in GER, the solid coal rib can fail in two primary manners: tensile failure and shear failure. In cases where the coal seam is hard and subjected to high stress from the rotation of the main roof, primarily tensile fracture failure occurs within the solid coal rib (refer to Fig. 10(a)). Conversely, when the coal seam is soft, shear stress surpasses shear strength resulting in shear sliding failure within the solid coal rib (refer to Fig. 10(b)). Therefore, effective control of the solid coal rib in GER involves preventing both tensile deformation and shear deformation. This aids in minimizing displacement of the solid coal rib, maintaining a relatively stable support point for overlying rock structure, and reducing intensity of overlying rock activity.

Given the considerable deformability of solid coal ribs, their deformation often proves to be unmanageable. While support systems can effectively mitigate and slow down surrounding rock deformation to some extent, they cannot completely prevent significant deformations from occurring. The failure of bolt (cable) support due to excessive deformation frequently results in the fracture of solid coal ribs. Therefore, it becomes imperative to apply adequate lateral support resistance on solid coal ribs in order to hinder loosening and compression of shallow coal bodies, minimize expansion and deformation of solid coal ribs, and prevent bolt (cable) anchorage failures. The determination of support resistance for bolt (cable)-supported solid coal ribs can be achieved using the following formula:

(26) $$p_{x}\geq \left[p_{0}\right]$$

In Eq. (26), *p*_x_ is the support resistance of the solid coal rib supported by bolt (cable); [*p*_0_] is the minimum necessary support resistance of the solid coal rib.

### 4.2 Determination of the support resistance of plastic solid coal rib to maintain stability

**Fig. 11:**
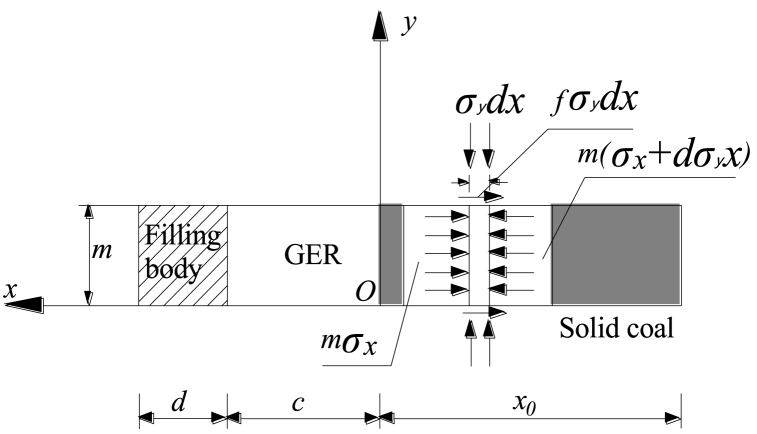
Limit stress state model of the solid coal rib in GER.

The stress concentration and plastic failure of the solid coal rib in GER occur simultaneously due to the rotation and sinking of the main roof. Moreover, cracks gradually form within the solid coal, progressively extending deeper. These crack formations result in fractures within the shallow coal, leading to a reduction in its bearing capacity. It is important to note that under extreme scenarios, the anchor structure of the solid coal rib falls within the crushing zone where there is no cohesion at the interface between the solid coal and both roof and floor surfaces. The limit stress state model depicted in Fig. 11 serves as an established representation for this phenomenon.

The limit equilibrium equation is established as follows:

(27) $$m\left(\sigma_{x}+d\sigma_{x}\right)-m\sigma_{x}-2\sigma_{y}fdx=0$$

In Eq. (27), *f* is the friction coefficient between roof and floor and coal seam.

According to the conditions of limit equilibrium zone, it is obtained as follows:

(28) $$\sigma_{y}=R_{c}+\frac{1+\sin\;\varphi }{1-\sin\;\varphi }\sigma_{x}$$

Taking the derivative on both sides, it is obtained as follows:

(29) $$\frac{d\sigma_{y}}{d\sigma_{x}}=\frac{1+\sin\;\varphi }{1-\sin\;\varphi }$$

Substituting Eq. (29) into Eq. (27) for solution,

(30) $$\ln\;\sigma_{y}=\frac{2fx}{m}\left(\frac{1+\sin\;\varphi }{1-\sin\;\varphi }\right)+D$$

According to the boundary conditions (*x* = 0, *σ*_*y*_ = *N*_0_), it is obtained as follows:

$$D=\ln\;N_{0}$$

Then,

(31) $$\sigma_{y}=N_{0}e^{\frac{2fx}{m}\left(\frac{1+\sin\;\varphi }{1-\sin\;\varphi }\right)}$$

In Eq. (31), *N*_0_ is the support capacity of solid coal rib, it is obtained as follows:

$$N_{0}=\frac{1+\sin\;\varphi^{*}}{1-\sin\;\varphi^{*}}p_{x}+R_{mc}^{*}$$

Where, $\varphi^{*}$ is the friction angle of residual strength of the solid coal rib; $R_{mc}^{*}$ is the residual compressive strength of the solid coal rib.

Since the surrounding rock is broken in the shallow part of the solid coal rib, its cohesion is approximately 0, that is $R_{mc}^{*}$ = 0. Therefore, the stress state in the anchor body is as follows:

(32) $$\begin{array}{l} \sigma_{x}=p_{x}e^{\frac{2fx}{m}\left(\frac{1+\sin\;\varphi^{*}}{1-\sin\;\varphi^{*}}\right)}\\ \sigma_{y}=p_{x}\frac{1+\sin\;\varphi^{*}}{1-\sin\;\varphi^{*}}e^{\frac{2fx}{m}\left(\frac{1+\sin\;\varphi^{*}}{1-\sin\;\varphi^{*}}\right)} \end{array}\}$$

If the average vertical load on the anchor is $\bar{\sigma }$, the support resistance of the solid coal rib shall reach:

(33) $$p_{x}=\bar{\sigma }\frac{1－\sin\;\varphi^{*}}{1＋\sin\;\varphi^{*}}e^{－\frac{2fx}{m}\left(\frac{1+\sin\;\varphi^{*}}{1-\sin\;\varphi^{*}}\right)}$$

Therefore, the support resistance of solid coal rib bolt (cable) is not lower than Eq. (33), so as to meet the stability of solid coal rib in plastic state.

**Fig. 12:**
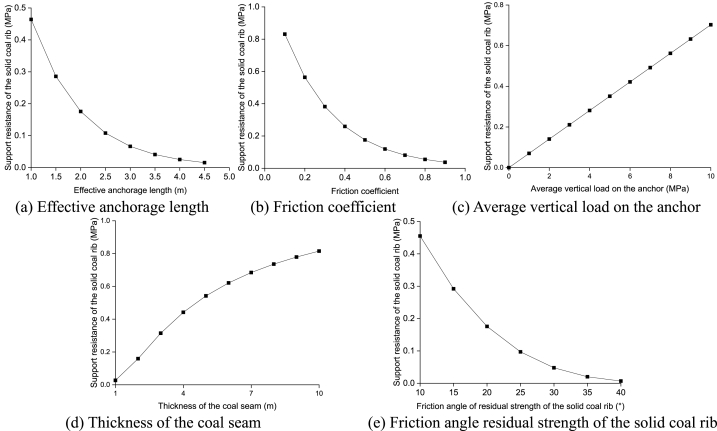
Relationship between the support resistance of the solid coal rib and these factors.

According to the geological and mining conditions of the 94103 working face in Qishan Coal Mine, the support resistance of the solid coal rib in GER is calculated using a single variable method. The relevant influencing factors include: effective anchorage length of bolt (cable) (reference value: 2.0 m), average vertical load on the anchor (reference value: 2.5 MPa), friction angle of residual strength of the solid coal rib (reference value: 20°), thickness of the coal seam (reference value: 2.1 m), and friction coefficient between roof, floor, and coal seam (reference value: 0.5). Fig. 12 illustrates the relationship between these factors and the support resistance of the solid coal rib.

As can be seen from Fig. 12 (a)–(e), the stability of plastic solid coal ribs depends on a variety of factors. The support resistance, which plays a crucial role in maintaining this stability, exhibits a negative exponential correlation with the effective anchorage length of bolt (cable). Additionally, it demonstrates a cubic relationship with the friction coefficient between the coal seam and both the roof and floor. Furthermore, there is a linear association with the average stress of the immediate roof, while a quadratic connection exists with both the thickness of the coal seam and residual friction angle of the solid coal rib. As these factors change, so does the support resistance required to sustain stability. Specifically, an increase in effective anchorage length of bolt (cable), friction coefficient between coal seam and roof/floor or residual friction angle leads to a decrease in support resistance needed. Conversely, an increase in average stress of immediate roof or coal seam thickness results in an increase in necessary support resistance.

### 4.3 Control countermeasures of the plastic solid coal rib in deep GER

Therefore, to enhance the stability of the solid coal rib, the following measures can be implemented.

(i) In the initial excavation of the roadway, it is recommended to utilize high-strength bolts and anchor cables for supporting the solid coal rib. This will improve both peak strength and residual strength of surrounding rock in the anchorage area, thereby retarding expansion of crushing and plastic areas. Consequently, this will increase stability of the plastic solid coal rib [39,40].
(ii) Consider using early strength materials with rapid resistance increasing properties for constructing filling body [10]. By doing so, load sharing between filling body and solid coal rib can occur at an earlier stage. As a result, rotation and sinking angle of immediate roof can be reduced which ultimately contributes towards enhancing stability of solid coal rib.
(iii) During mining operations, reinforce support with high resistance to alleviate pressure on solid coal rib. This measure aids in improving stability.
(iv) Employ inclined arrangement of bolts (anchor cables) at top and bottom corners of solid coal rib to enhance mechanical properties at interface between solid coal rib and roof/floor. This arrangement promotes increased stability.
(v) Whenever possible without compromising retained roadway usage requirements, minimize roadway height as it helps augmenting stability of solid coal rib.
(vi) Determine appropriate bolt support parameters. In cases where broken area depth exceeds anchor bolt's load-bearing capacity foundation limit consider adopting shotcrete and grouting reinforcement techniques to strengthen bearing capacity of a solid coal rib. Such reinforcement measures are beneficial for increasing overall stability.

## 5 Deformation mechanism of plastic solid coal rib in deep GER

### 5.1 Solid coal rib plastic zone distribution subjected to filling body width and retained roadway width

**Fig. 13:**
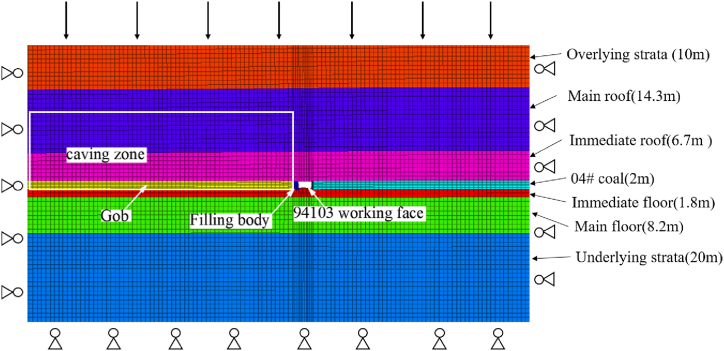
Numerical calculation model of 94103 working face.

Based on the experimental conditions of the working face (94103) in Qishan Coal Mine and the mechanical properties derived from laboratory tests conducted on high-water materials, a numerical calculation model was established. The diagram in Fig. 13 illustrates the dimensions of the model, which measures 154.8 m in length, 2 m in width, and 63 m in height. It consists of a total of 27,984 cells and 42,813 nodes. The displacement at the bottom of the model is fixed, while limitations are imposed on normal displacements at both front and rear boundaries. To simulate coal rock mass behavior, we employed the Mohr-Coulomb constitutive model; for gob simulation (as depicted by Fig. 13), we utilized a double-yield constitutive model; finally, for filling body and anchored solid coal rib simulation [[41], [42], [43], [44]], we adopted a strain-softening constitutive model. Mechanical parameters necessary for modeling were obtained through laboratory testing as well as numerical inversion techniques (refer to Table 2, Table 3, and Table 4).

**Table 2:** Numerical calculation parameters of 94103 working face.

Rock mass	Thickness/m	Elastic modulus/GPa	Poisson ratio	Friction/°	Cohesion/MPa	Tension/MPa
Overlying strata	10	5.02	0.25	34	3.0	0.8
Main roof	14.3	5.92	0.23	24	2.52	1.0
Immediate roof	6.7	4.03	0.26	20	1.33	0.8
04# coal	2.0	2.57	0.29	20	4.46	1.3
Immediate floor	1.8	4.13	0.29	16	0.75	0.8
Main floor	8.2	10.02	0.22	42	3.26	1.4
Underlying strata	20	5.02	0.25	28	1.93	1.2

**Table 3:** Numerical calculation quantitative parameters of strain-softening constitutive model of coal rib.

Category	Elastic modulus/GPa	Poisson ratio	Friction/°	Tensile strength/MPa
Value	2.57	0.29	20	1.3

**Table 4:** Numerical calculation variable parameters of strain-softening constitutive model of coal rib.

Plastic parameter	Cohesion/MPa	Plastic parameter	Cohesion/MPa	Plastic parameter	Cohesion/MPa
0	4.46	0.08	4.16	0.2	3.26
0.02	4.46	0.1	3.86	0.3	2.46
0.04	4.46	0.12	3.86	1	2.46
0.06	4.16	0.14	3.86		

**Fig. 14:**
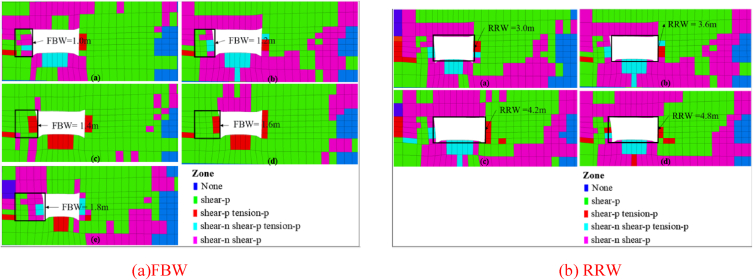
Plastic zone distribution characteristics of the solid coal rib subjected to different FBW and RRW.

**Fig. 15:**
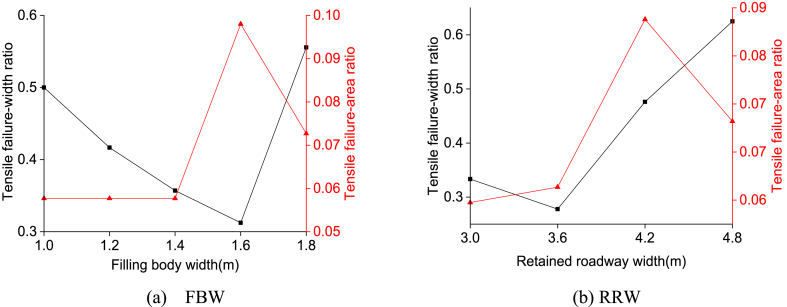
Tensile failure-width ratio and tensile failure-area ratio subjected to different FBW and RRW.

Different numerical calculation schemes were proposed for a range of filling body widths (FBWs) between 1.0 m and 1.8 m, as well as retained roadway widths (RRWs) between 3.0 m and 4.8 m respectively. Fig. 14 visually demonstrates how plastic zones distribute within solid coal ribs when subjected to varying FBWs and RRWs. The ratio between coal rib's width at which it experiences tensile failure with respect to either FBWs or RRWs is defined as "tensile failure-width ratio". Additionally, "tensile failure-area ratio" refers to the proportion between tensile failure area on a coal rib compared with overall failing region. Statistical outcomes illustrating these ratios across diverse combinations of FBWs and RRWs can be found in Fig. 15.

As can be seen from Fig. 14(a)–(b) and Fig. 15(a)-(b), the ratio of tensile failure-width for the solid coal rib initially exhibits a decreasing trend, followed by an increasing trend with an increase in FBW. Similarly, the ratio of tensile failure-area demonstrates an initial increase, followed by a decrease as FBW increases. At an FBW of 1.6 m, the minimum value for the tensile failure-width ratio is 0.31, while the maximum value for the tensile failure-area ratio is 0.098. Beyond an FBW of 1.6 m, there is a noticeable escalation in tension-induced failures within the solid coal rib, resulting in its extension. When considering RRW, it can be observed that the ratio of tensile failure-width for the solid coal rib follows a similar pattern as before - initially decreasing and then increasing with an increase in RRW; whereas the ratio of tensile failure-area shows an initial increase followed by a subsequent decrease with increasing RRW values. The minimum value for the tensile failure-width ratio occurs at an RRW of 3.6 m (0.28), while at an RRW of 4.2 m (0.093), we observe its maximum value for the tensile failure-area ratio to occur. For RRWs greater than 4.2 m, there is a significant rise in tension-induced failures within the solid coal rib leading to its extension.

### 5.2 Solid coal rib plastic zone distribution subjected to rock bolt support schemes

**Fig. 16:**
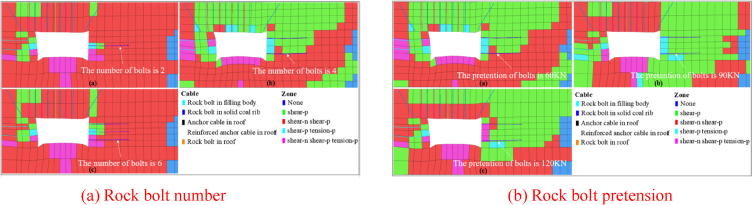
Solid coal rib plastic zone distribution subjected to different rock bolt support schemes.

**Fig. 17:**
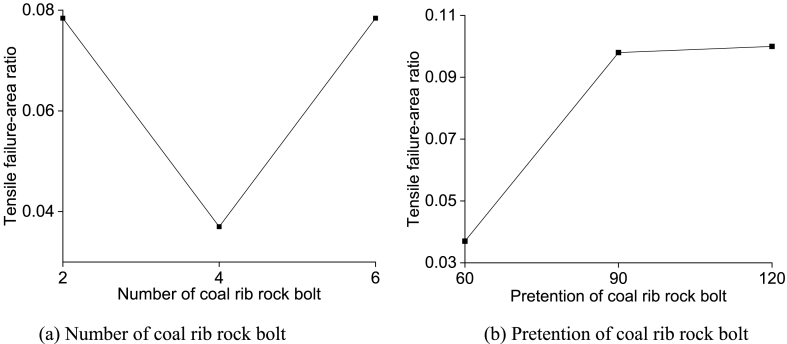
Statistical results of tensile failure-area ratio subjected to rock bolt number and pretension.

To evaluate the solid coal rib plastic zone distribution subjected to different rock bolt support schemes, several numerical simulation schemes with different rock bolt number on the coal rib (n = 2,4,6) and different rock bolt pretension (60 kN, 90 kN, 120 kN) was put forward. The solid coal rib plastic zone distribution subjected to different rock bolt support scheme is shown in Fig. 16. The statistical results of tensile failure-area ratio subjected to rock bolt number and pretension are shown in Fig. 17.

It can be seen from Fig. 16(a)–(b) and Fig. 17(a)-(b) that the tensile failure-area ratio of the solid coal rib significantly decreases when 4 rock bolts are placed on the coal rib, which is 52.8 % lower than that of 2 and 6 rock bolts. When the rock bolt pretension increases from 60 kN to 90 kN, the tensile failure-area ratio of the solid coal rib increases obviously. When the rock bolt pretension is 90 kN, the tensile failure-area ratio is 0.098, and then the tensile failure-area ratio tends to be stable with the increase of the coal rib rock bolt pretension. With the increase of the rock bolt pretension, the tensile failure tends to extend to the coal rib.

## 6 Engineering practice

### 6.1 Control countermeasures design of GER

Based on the findings of the aforementioned research, the primary focus of controlling goaf edge roadway (GER) involves implementing measures related to the roof, solid coal rib, and filling body.

#### 6.1.1 The roof reinforcement design

**Fig. 18:**
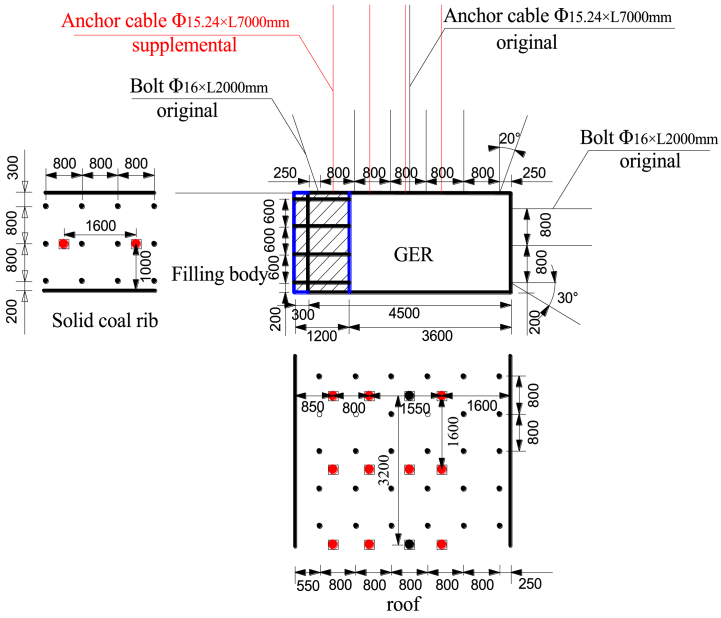
Reinforcement design of 94103 haulage gate (unit: mm).

To reinforce the 94103 haulage gate, a series of anchor cables are positioned at distances of 1550 mm, 2350 mm, 3150 mm, and 3950 mm from the solid coal rib. The spacing between these anchor cables is set at intervals of 1600 mm, 3200 mm, 1600 mm, and 1600 mm respectively. Each anchor cable has a diameter of 15.24 mm and a length of 7 m. Fig. 18 illustrates the reinforcement design for this particular haulage gate.

#### 6.1.2 The solid coal rib reinforcement design

According to Eq. (33) and the geological and mining conditions, the minimum support resistance of the solid coal rib in 94103 working face is 0.214 MPa. Hence, a row of anchor cables is set at the solid coal rib 1000 mm away from the retained roadway floor, and the spacing of anchor cables are 1600 mm. The diameter of anchor cable is 15.24 mm and the length is 4 m.

Considering Eq. (33) alongside geological and mining conditions specific to this area, it is determined that a minimum support resistance value of approximately 0.214 MPa is required for the solid coal rib in working face number "94103". Consequently, a row of anchor cables is installed along the solid coal rib with a distance measurement of 1000 mm from the retained roadway floor. The spacing between these anchor cables remains consistent at an interval of 1600 mm. The diameter foreach anchor cableis15.24mmand their respective lengths measure4m.

#### 6.1.3 The filling body design

**Fig. 19:**
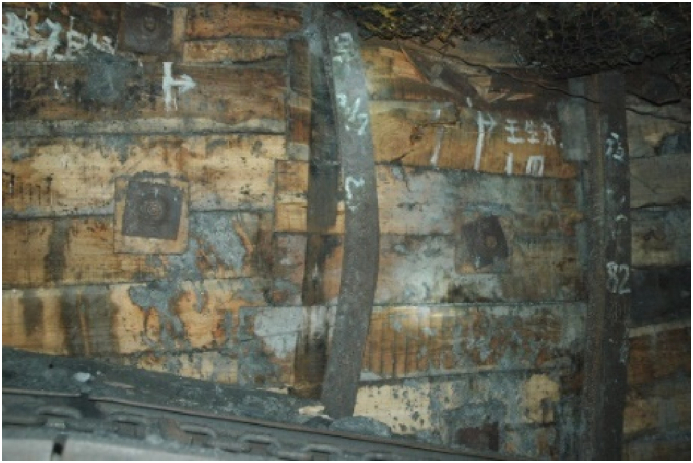
In-situ filling body.

The filling body design for roadside construction incorporates cement, gangue, coal, and water as materials. This material combination ensures the fulfillment of normal GER requirements with a reasonable ratio. Through laboratory testing and numerical calculations, it has been determined that the water-cement ratio for roadside filling materials in Qishan Coal Mine is 30 %, with a cement content of 33 %. The ratio employed is 0.43:1:1, while the actual width of the filling body measures 1.2 m. Coal gangue serves as the primary coarse aggregate in this design. The density values are as follows: cement - 3 × 103 kg/m3, coal - 1.25 × 103 kg/m3, and gangue - 1.8 × 103 kg/m3.Specifically, Portland cement type 425 is utilized in this project. To ensure accurate testing results considering volume effects on test blocks, raw coal and gangue are obtained from No.9 coal seems underground working face before being crushed and screened to achieve a maximum particle size of 5 mm. For protection purposes, both internal and external sides of the filling body are safeguarded using an I-steel profile (11#). Each steel piece measures at a length of 2200 mm with row spacing set at intervals of1000 mm. In terms of side protection points within the old pond area surrounding the filling body structure, log (or semi-log) materials can be utilized instead if desired or necessary. Furthermore, two recovered old bolts are embedded between two I-shaped steels to enhance stability. The spacing between anchor bolts and rows is set at600 × 1000 mm.Fig. 19 provides an illustration showcasing the in-situ filling body configuration.

### 6.2 Practice effect

**Fig. 20:**
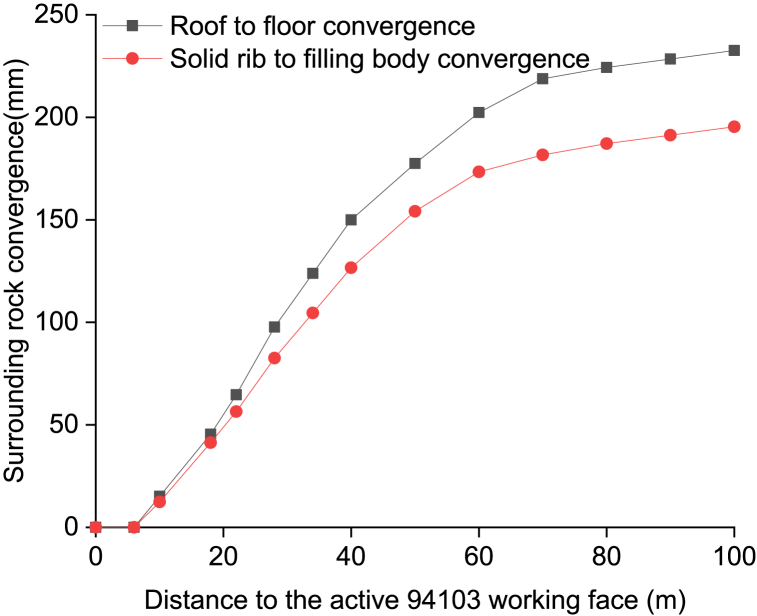
Surrounding rock convergence behind the active 94103 working face.

The convergence of the surrounding rock behind the active 94103 working face, measured using the cross-point method, is illustrated in Fig. 20. Overall, effective measures have been implemented to control deformation in the surrounding rock and maintain the integrity of the solid coal rib. The convergence between roof and floor is recorded as 232 mm, while that between solid coal rib and filling body measures at 190 mm. Additionally, it should be noted that the retained roadway satisfactorily meets both ventilation and pedestrian requirements.

## 7 Conclusions

(1) Due to the rotation and sink of the two lateral broken blocks of the main roof, the shallow part of the solid coal rib of the retained roadway often enters the plastic state and has poor stability, which is prone to instability and failure subjected to loading and unloading from mining-induced stress.
(2) The mechanical properties of the solid coal rib have an important influence on the second lateral break position of the immediate roof and the stability of key blocks. With the improvement of the solid coal rib support resistance and mechanical properties (UCS), the second break position of the key block is gradually away from the solid coal rib. The sliding instability coefficient *K*_1_ decreases with the increase of the solid coal rib support strength, indicating that the larger solid coal rib support strength is conducive to the stability of key block B. The sliding instability coefficient *K*_1_ first decreases and then increases with the increase of the UCS of the solid coal rib, and the extrusion deformation instability coefficient *K*_2_ increases with the increase of the UCS of the solid coal rib, indicating that there is a certain limit of the support strength of the solid coal rib.
(3) Taking the GER of 94103 working face in Qishan Coal Mine of Xuzhou Mining Bureau as an engineering case, the mechanical model of the solid coal rib supported by bolts/cables in deep GER is established, and it is obtained that the required bolts/cables support resistance for the stability of the plastic solid coal rib is 0.214 MPa.
(4) When the FBW is 1.6 m, and the tensile failure-width ratio is the minimum and the tensile failure-area ratio is the maximum. When the RRW is 3.6 m, the tensile failure-width ratio is the minimum. When the RRW is 4.2 m, the tensile failure-area ratio is the maximum. When the number of rock bolts placed in the solid coal rib is 4 and the rock bolt pretension isn't less than 90 kN, the tensile failure-area ratio is stable and the solidcoal rib deformation is small.
(5) According to the failure mechanism of the plastic solid coal rib in deep GER, the surrounding rock control countermeasures of the retained roadway in 94103 working face are determined. The field monitoring results show that the solid coal rib reinforced by bolts and anchor cables can remain stable, and the surrounding rock deformation of the retained roadway can meet the production requirements.

## Data availability

Data related to your research is not stored in publicly available repositories. The datasets used or analyzed during the current study are available from the corresponding author on reasonable request.

## Additional information

No additional information is available for this paper.

## CRediT authorship contribution statement

**Shiqiang Xu:** Writing – original draft, Data curation. **Zizheng Zhang:** Writing – review & editing, Project administration, Funding acquisition. **Jinlin Xin:** Writing – review & editing, Software, Investigation. **Jianbiao Bai:** Supervision, Project administration, Funding acquisition. **Xingen Ma:** Software, Resources. **Ren Zhai:** Resources. **Wenda Wu:** Writing – review & editing.

## Declaration of competing interest

The authors declare that they have no known competing financial interests or personal relationships that could have appeared to influence the work reported in this paper.
